# Efficacy of graphene oxide-loaded cationic antimicrobial peptide AWRK6 on the neutralization of endotoxin activity and in the treatment of sepsis

**DOI:** 10.18632/aging.203397

**Published:** 2021-08-13

**Authors:** Bo Song, Hongli Zhao, Haiyan Yang, Shengji Wang

**Affiliations:** 1 Department of Emergency, Yantaishan Hospital, Yantai, Shandong Province, China; 2 Department of Senile Diseases, Dongying City Shengli Hospital, Dongying, Shandong Province, China; 3 Department of Emergency, Linyi People’s Hospital, Linyi, Shandong Province, China

**Keywords:** graphene, AWRK6, AWRK6/GO, septicemia, endotoxin

## Abstract

Objective: This study is to assess the therapeutic effect of graphene oxide (GO) loaded with AWRK6 on endotoxin-induced sepsis.

Method: AWRK6/GO was prepared by GO loaded AWRK6, with the structure characterization of AWRK6/GO conducted by atomic force microscope (AFM) and ultraviolet spectrophotometer, the sustained release rate of AWRK6/GO detected by high performance liquid chromatography (HPLC), and the neutralization ability of AWRK6/GO to lipopolysaccharide (LPS) tested by *in vitro* experiments. The levels of IL-8 and TNF-α in mouse cells after drug intervention were detected by ELISA; a LPS mouse model was established to observe the effects of drug intervention on the survival cycle and survival rate of mice.

Results: The sustained drug release rate of AWRK6/GO reached 85% within 24 hours observed under *in vitro* conditions, with an efficient neutralization effect to LPS (*P* < 0.01); Compared with the control group, the intervention of LPS succeeded in remarkably elevating the levels of IL-8 and TNF-α in the whole blood and macrophages of the mice (*P* < 0.01), whose survival cycle and survival rate consequently observed an obvious decline (*P* < 0.01); The intervention with AWRK6 or AWRK6/GO predominantly brought down the levels of IL-8 and TNF-α in the whole blood and macrophages of mice given LPS (*P* < 0.01), resulting in an elevation of the survival rate and survival time (*P* < 0.01).

Conclusion: GO loaded with cationic antimicrobial peptide AWRK6 exerts a rosy neutralization effect on endotoxin activity, with no obvious side effects on mice observed, which is of certain application value in the treatment of sepsis.

## INTRODUCTION

To date, the treatment of sepsis, a disease of host immune disorders caused by bacterial infection, an array of therapeutic targets applied in clinical practice notwithstanding, still fails to yield an ideal outcome [1, 2]. Endotoxin, also known as LPS, is an important component of the cell wall of Gram-negative bacteria and an essential inflammatory factor in the body [3]. Studies have confirmed that a small amount of endotoxin can induce a series of inflammatory reactions in the human body, leading to septic shock or even death of patients in severe cases [4]. Hence, sepsis triggered by endotoxin has become a world-wide medical challenge which consequently requires an excavation of a therapeutic agent that can ensure a substantial interception of the stimulation from LPS to the body’s immune system.

Antibacterial peptides, a key part of the body’s immune defense with disadvantages to the drug-resistance of pathogenic bacteria, can eliminate the pathogens by the disruption of the cell membrane in direct interaction and the regulation with the host immune system [5, 6]. Recent studies have shown that antimicrobial peptides intervention, long appreciated by its excellent anti-inflammatory effects on the substantial inhibition of endotoxin activity in bacteria extermination and its efficacy of diminishing the production of pro-inflammatory cytokines by inhibiting the activation of monocytes and macrophages [7, 8], has therefore become a promising antimicrobial strategy in clinical practice. AWRK6 (SWVGKHGKKFGLKKHKKH) is a new type of cationic antibacterial peptide, which is transformed from the antibacterial peptide dybowskin-2CDYa (SAVGRHGRRFGLRKHRKH) with strong antibacterial activity [9]. Graphene, the thinnest nanomaterial with only one atom thickness, is the basic unit of many nanomaterials [10]. It has been proven by in-depth research to have a wide range of applications in many fields such as medicine and chemical engineering [11]. GO exerts a tremendous fascination on the academia with its excellent biocompatibility, a stable dispersity in an aqueous solution, and a large specific surface area that can be used to load small molecule drugs [12, 13]. To elevate the inhibition effect of AWRK6 on LPS, this study focuses on graphene nanoparticles loaded with AWRK6, and evaluates its effect in neutralizing endotoxin activity and treating sepsis, with an aim to provide a reference for the treatment of sepsis in the clinic.

## RESULTS

### Structural characterization of AWRK6/GO

**Figure 1:**
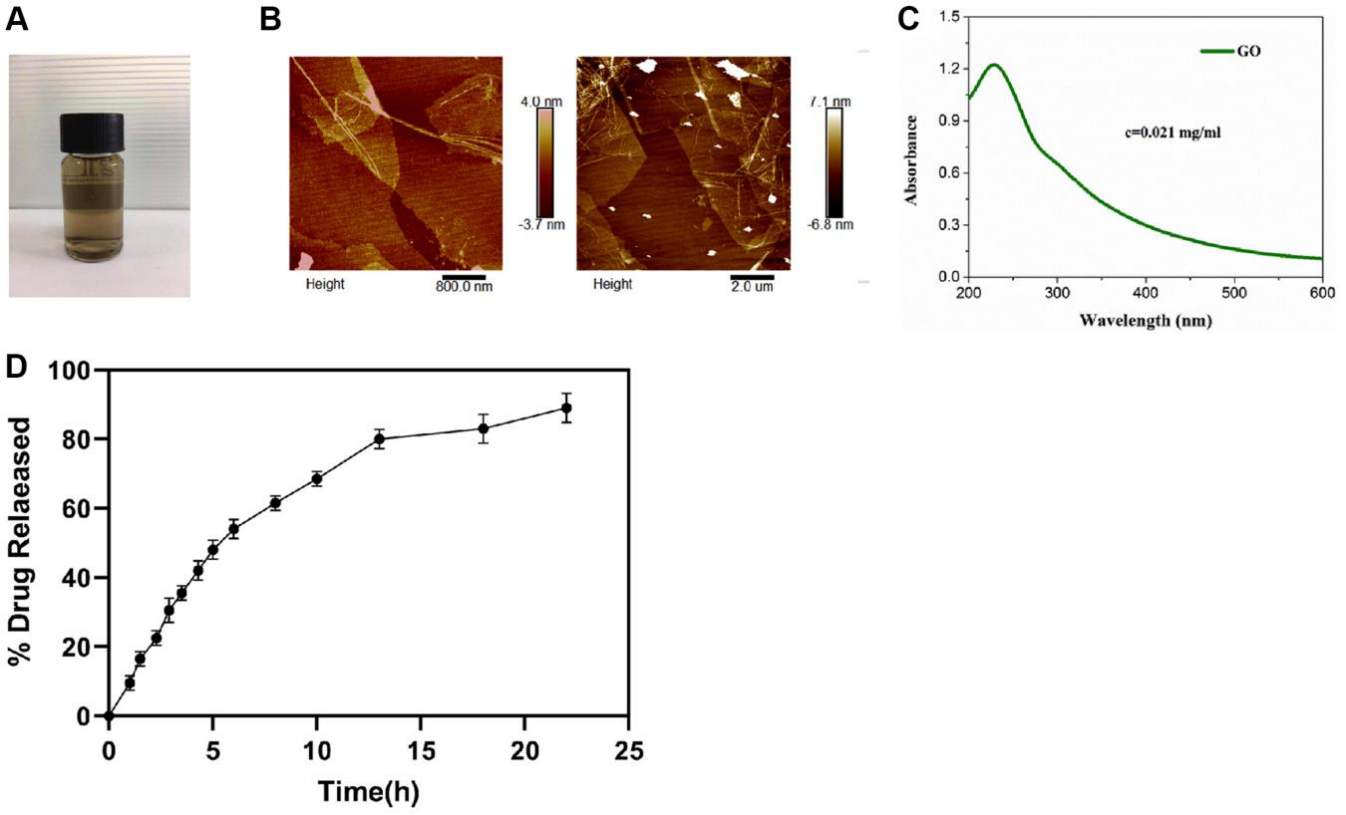
**AWRK6/GO characterization and drug release rate (A–D).** Note: (**A**) AWRK6/GO sample; (**B**) AWRK6/GO.

The AWRK6/GO AFM characterization, UV characterization, and drug release rate changes were shown in Figure 1A–1D. Closer inspection of Figure 1 presented a remarkable sustained release rate of AWRK6/GO 85% within 22 hours. Meanwhile, we identified the half-life of AWRK6/GO was 720 minutes.

### The inhibitory effect of AWRK6/GO on LPS activity *in vitro*

**Figure 2:**
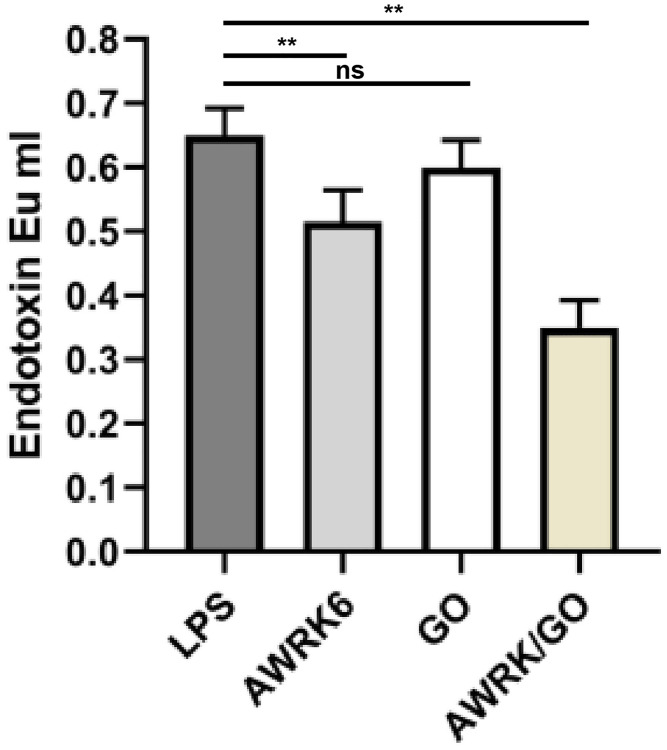
Inhibitory effect of AWRK6/GO on LPS activity in *in vitro* experiments.

The results from Figure 2 showed that compared with the LPS control group, the LPS concentration in the GO group observed no obvious changes (*P* > 0.05), while the LPS concentration in the AWRK6 or AWRK6/GO group obtained an apparent decrease (*P* < 0.01), in which a greater fall of the LPS level in the AWRK6/GO group was found (*P* < 0.01).

### The effect of AWRK6/GO intervention on the levels of inflammatory factors in the whole blood and macrophages of LPS mice

**Figure 3:**
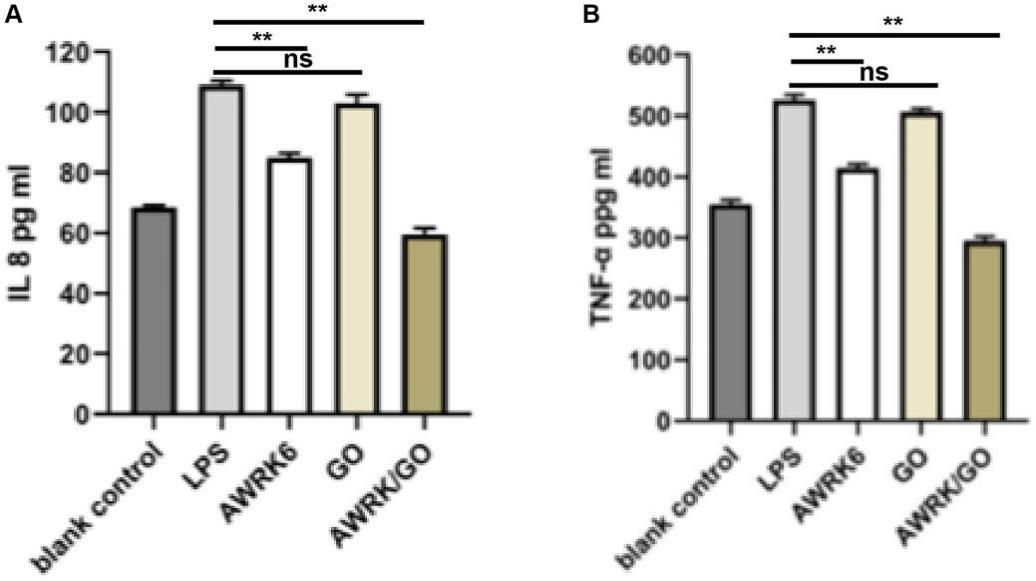
**The effect of AWRK6/GO intervention on the levels of inflammatory factors in the whole blood and macrophages of LPS mice.** Note: (**A**) IL-8 expression level; (**B**) TNF-α level.

The results in Figure 3A, 3B showed that compared with the control group, the serum levels of inflammatory factors IL-8 and TNF-α in the mice treated with LPS were notably increased (*P* < 0.01); By contrast to the LPS control group, no evidence of apparent changes in the serum levels of IL-8 and TNF-α in the GO group was detected (*P* > 0.05). AWRK6 or AWRK6/GO yielded a markedly decline of the serum levels of IL-8 and TNF-α in mice (*P* < 0.01), in which AWRK6/GO garnered a more prominent result (*P* < 0.01).

### The effect of AWRK6/GO intervention on the prognosis and survival of LPS mice

**Figure 4:**
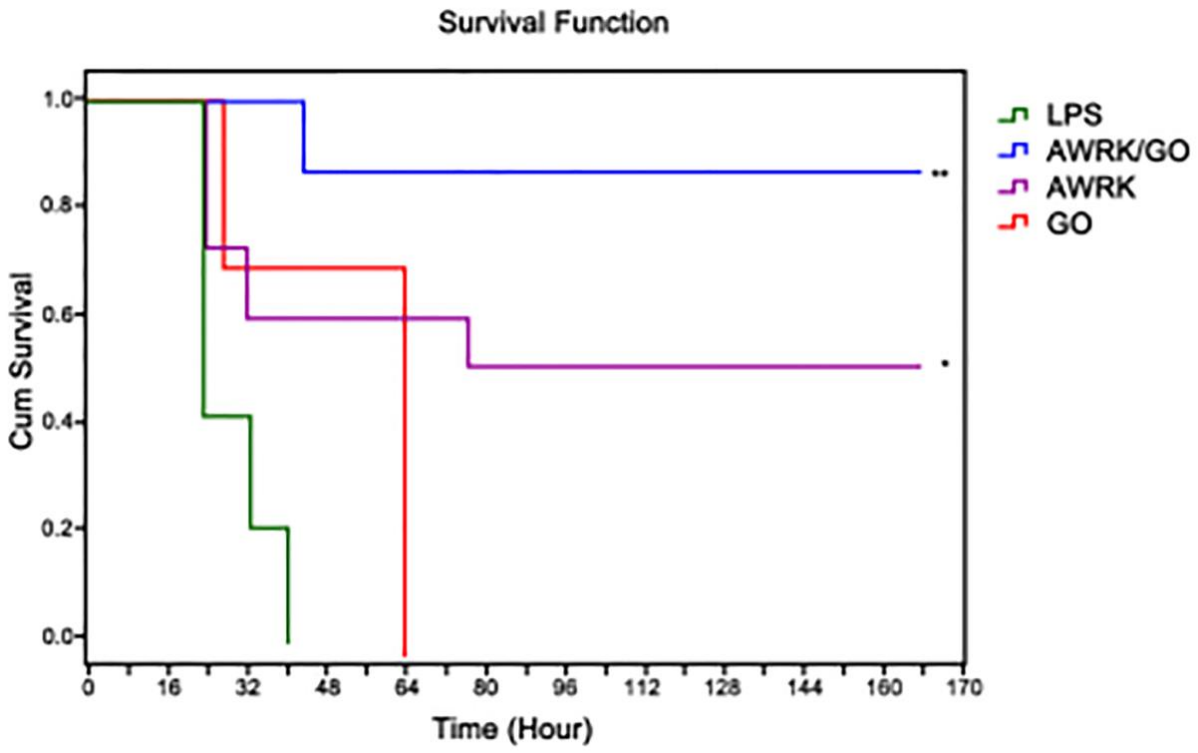
The effect of AWRK6/GO intervention on the prognosis and survival of LPS mice.

All mice were injected with a lethal dose of 0.5 mL of LPS (50 mg/kg). In the LPS control group, all mice died within 40 hours compared with the 56 hours of the GO intervention group. However, the group with AWRK6 or AWRK6/GO intervention successfully extended the survival time to over 168 hours. In comparison with mice in the LPS control group, no significant changes were detected in terms of the survival period and survival rate of the mice treated with GO (*P* > 0.05), while the AWRK6 or AWRK6/GO yielded a desirable outcome regarding the long-term survival rate and survival rate of the mice (*P* < 0.01), in which AWRK6/GO witnessed a greater increase than AWRK6 (*P* < 0.01). See Figure 4.

### The effect of AWRK6/GO on the activity of liver cells, spleen cells and macrophages in LPS mice

**Figure 5:**
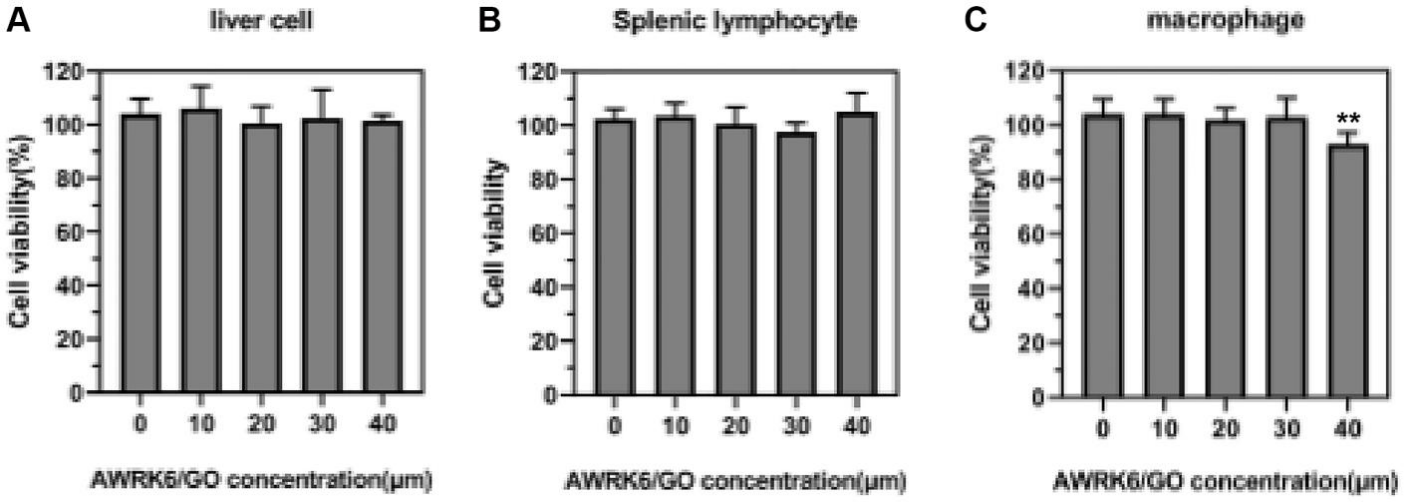
**The effect of AWRK6/GO intervention on the activity of liver cells, spleen cells and macrophages in mice.** Note: (**A**) Liver cell activity; (**B**) Spleen cell activity; (**C**) Macrophage activity.

The results in Figure 5A–5C showed that compared with the control group, 10, 20 and 30 μg/mL AWRK6/GO exerted no significant inhibitory effect on the activity of mouse liver cells and lymphocytes (*P* > 0.05), while the activity of macrophages was suppressed when the AWRK6/GO concentration reached to 40 μg/mL (*P* < 0.05).

### The effect of AWRK6/GO on mouse organs and tissues

**Figure 6:**
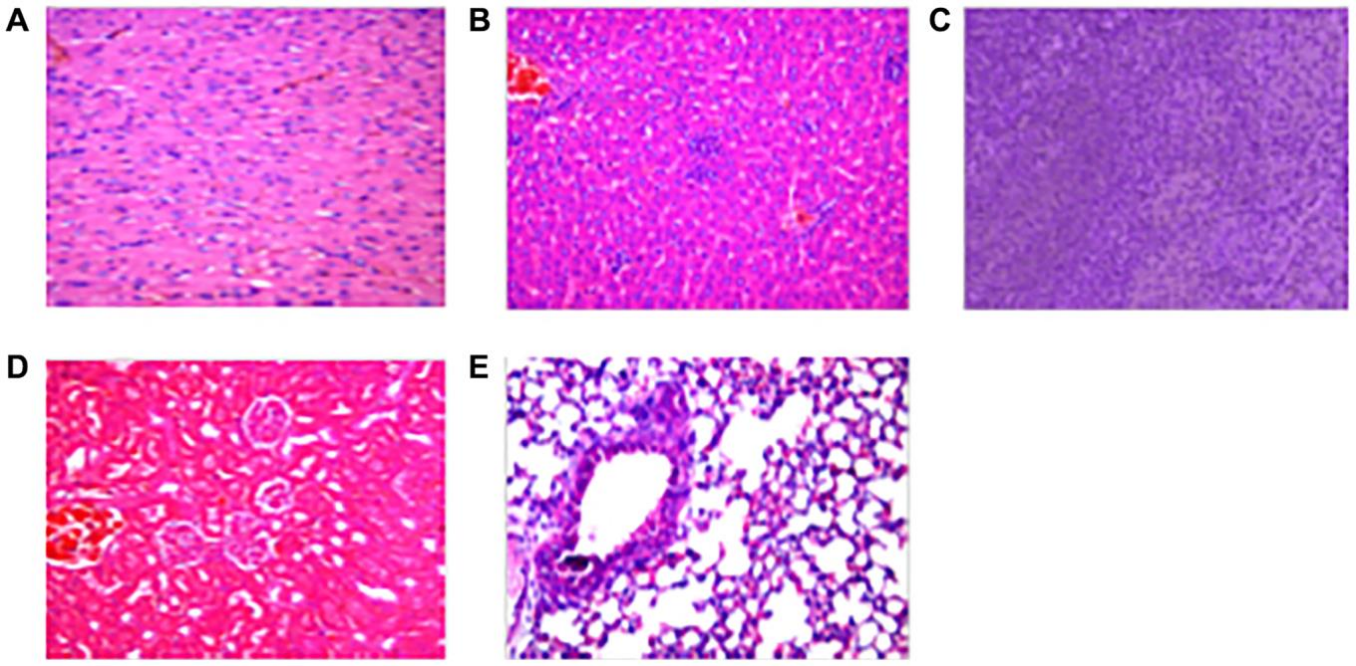
**The effect of AWRK6/GO on mouse organs and tissues.** Note: (**A**) Heart; (**B**) Liver; (**C**) Spleen; (**D**) Kidney; (**E**) Kidney.

The effects of AWRK6/GO on mouse heart, liver, spleen, lung and kidney tissues were shown in Figure 6A–6E, which was indicative of a rather hidden toxic effects of AWRK6/GO on the organs of mice (*P* > 0.05).

## DISCUSSION

As one of the perilous diseases usually induced by bacterial infection, sepsis invariably gives rise to fever, joint swelling and pain, and systemic inflammation [14]. Endotoxin is one of the key substances that leads to the patient’s immune response by bacteria through invading macrophages and neutrophils, which is considered as one of the major causes of sepsis [15]. Studies have found that the binding of LPS and the TLR4 protein on the cell surface may induce cells to produce a large number of inflammatory factors [16]. At present, antibiotics are often used clinically to inhibit bacteria in patients with sepsis, though its neutralization effect on endotoxin toxicity were rather concealed [17]. That a predominant neutralization effect of AWRK6 antimicrobial peptides on the toxicity of LPS is able to mitigate the inflammatory response of cells triggered by LPS has also been reported. AWRK6 is a new type of cationic antibacterial peptide, possessing an α-helical structure and an affinity for LPS [18]. This study targeted the therapeutic effect of AWRK6 on LPS-induced sepsis, and evaluated the application value of GO-loaded AWRK6 in the treatment of sepsis.

GO is a new type of active material that contains a large number of oxygen-containing functional groups, characterized by a positive biological activity, an excellent hydrophilicity and a stable dispersity in water, which is widely used in the chemical and medical areas [19]. Biodistribution analysis revealed that GO presented in mice brain, spleen, liver, kidney and bone marrow within a month after injection [20]. Eventually, GO materials were mostly excreted through urinary system, whereas a small portion of GO would be sequestered by spleen [21, 22]. However, no detectable pathological consequences in the spleen were found to be caused by this GO accumulation [22]. In addition, analyses using both bright-field TEM coupled with electron diffraction and Raman spectroscopy spotted *in vivo* intracellular biodegradation of GO, and the spectral features of GO crystals barely existed in spleen nine months after injection [22]. Meanwhile, GO is widely used in human. For example, it has been reported that Graphene and GO enhance ROS accumulation in human skin keratinocytes [23]. GO nanoplatelets improve anti-cancer effect of cisplatin on human lung cancer cells [24]. It has been found GO induces anti-angiogenic impact of GO in primary human endothelial cells [25].

It was found in this study that AWRK6/GO was more competent in the suppression of LPS activity in an *in vitro* environment than AWRK6, while LPS was barely affected by GO. A larger specific surface area enables GO to strongly absorb substances with a small molecular weight, indicating no evident inhibitory effect on LPS by GO. Compared with AWRK6, GO in AWRK6/GO elevates the drug's loading capacity, which further enhances the inhibitory effect of AWRK6 on LPS activity by increasing the possibility of interaction between AWRK6 and LPS. Several studies determined that GO could be engulfed by multiple cell types such as macrophages, natural killer cells and so on [26, 27]. GO could serve as an antioxidant to impair intracellular ROS accumulation, and attenuate inflammatory polarization to type II macrophages [26]. Another study suggested that nanoformulated GO could stimulate neutrophile activation via inducing ROS formation [28]. On the other hand, Chen and colleagues reported no obvious cytotoxicity or significant cellular uptake of GO in A549 lung cancer cells at low dose, while higher dose of GO induced oxidative stress and slightly impaired the cell viabilities [29]. These data demonstrated that the diverse doses and functionalization notably altered the internalization and effects of GO on various cell types. Our data suggested that only high dose of AWRK6/GO cause slightly retarded viability of macrophage, yet the internalization of AWRK6/GO by macrophage, leukocytes and normal liver cells, as the inflammatory response still need to be explored in future studies. Moreover, the pharmacokinetics (PK) and pharmacodynamics (PD) of GO were widely studied by previous studies. For example, Zhang et al., reported that the GO shows a long blood circulation time (half-time 5.3 ± 1.2 h) [30]. The presence of GO in bladder gradually increases within 1 hour after injection and reached the maximum concentration after 6 hours [31]. Li and colleagues compared the retention of GO and GO-PEG, and found that GO particles retained partially in lung and mostly in liver and spleen, while PEG coating improves the biocompatibility of GO, decreasing its retention in liver, lung, and spleen. Yet both GO and GO-PEG were still detectable 3 months after injection [32]. We will analyze the PK and PD of GO in our system in future investigations.

Sepsis is an inflammatory reaction that affects the patient’s systemic organs. A small amount of endotoxin can trigger a significant increase in the level of inflammatory factors in the body as it is highly sensitive to bacterial endotoxins [14]. IL-8 and TNF-α, produced by monocytes-macrophages, can promote the body’s inflammatory response and reflect the degree of inflammation, which are considered as vital indicators for the diagnosis of sepsis [33, 34]. Corresponding results discovered in this study that after LPS induction, the levels of IL-8 and TNF-α in mouse whole blood and macrophages observed a sharp rise with LPS induction but a notable decline after intervention with AWRK6 or AWRK6/GO. Moreover, prior studies proved that AWRK6 can significantly improve the levels of IL-8 and TNF-α in LPS mice, and inhibit body inflammation [34]. Extended results in this study confirmed that in comparison with AWRK6, AWRK6/GO prominently drove down the level of inflammation in mouse whole blood and macrophages, suggesting that AWRK6/GO can optimize the inflammatory response in mice by elevating the ability to neutralize LPS. Moreover, HMGB1 is a nuclear protein that could be released by macrophages at late phase of LPS-caused inflammatory response [35]. In this work, we detected the early inflammatory response under LPS induction, which was represented by the levels of IL-8 and TNF-α. The HMGB1 levels released to blood system or in tissues at later phase was not detected, and would be discussed in future study.

In this study, mice were injected with 50 mg/kg of LPS, and then AWRK6 or AWRK6/GO was used to evaluate the application value of AWRK6/GO in the treatment of sepsis. The results showed that compared with LPS control mice, both AWRK6 and AWRK6/GO garnered a favorable outcome in ameliorating the survival time and survival rate of LPS mice. It has been stated by Jin et al. [36] that AWRK6 has a protective bearing on LPS-induced liver cells in mice, and can inhibit the liver cell apoptosis induced by LPS in mice by participating in the regulation of the intracellular MAPK signaling pathway. That the improvement of drug efficacy was achieved by the loading of GO that has a low toxicity has been reported [37]. Zhang et al. [38] confirmed that the GO-loaded pro-apoptotic polypeptide drug delivery system effectively inhibit the proliferation of cancer cells. Similar results were reported in this study that AWRK6/GO was more efficient and effective with regard to the amelioration of the survival time and survival rate of LPS mice than AWRK6. Vivo experiments displayed a survival rate of mice as high as 90% in the AWRK6/GO group seven days after the injection of AWRK6/GO, which was apparently higher than those of other groups, indicating a strong neutralization effect of AWRK6/GO on LPS and an elevation of inhibitory effect of AWRK6 on LPS activity *in vivo*.

In conclusion, the AWRK6/GO complex formed by loading AWRK6 with GO can remarkably enhance the inhibitory effect of AWRK6 on LPS toxicity, prolong the life cycle of LPS mice, and elevate the survival rate of LPS mice with good biological safety.

## MATERIALS AND METHODS

### Preparation of GO

Preparation: Added concentrated sulfuric acid into the graphene, fully stirred, slowly added potassium permanganate, fully stirred for 20 minutes, and continued stirring in a 35°C constant temperature water bath until the solution turned green; moved the solution from the 35°C water bath to the ice water bath, continued stirring till purple red smoke appeared, then continue stirring for another 30 minutes. Subsequently, added 4 times the volume of cold water to the solution, continued stirring until the solution turned brown, and added cold water again until the foam disappeared and the solution turned orange-yellow. Rinsed the precipitate with 10% HCL, water and absolute ethanol, and stripped the graphene oxide into GO by the ultrasonic dispersion method and dried.

### Preparation and characterization of AWRK6/GO

Added 3 mg of GO into 20 mL of AWRK6 solution (20 mg/L) for a 160-minute reaction in a 30°C water bath shaker (130 revolutions/min). used hydrochloric acid and sodium hydroxide solution to adjust the pH value of the AWRK6 solution, filtered with a 0.45 μm water phase filter, and dissolved AWRK6/GO with methanol.

Characterization of AWRK6/GO: AWRK6/GO was characterized by AFM and ultraviolet-visible spectrophotometer.

### Drug release

The dialysis bag method was used to investigate the drug release rate *in vitro*. The GO nanoparticle solution loaded with AWRK6 was placed in 1 mL of PBS (pH 7.4 or pH 5.0) containing 0.1% Tween 80, put in a dialysis bag after it fully dispersed, and placed in a centrifugal tube containing 30 mL of the corresponding release medium. A vitro release test was performed in the shaker with 37°C and 100 rpm. 200 μL of release medium were taken after 0.5, 1, 2, 3, 4, 6, 8, 12, 24, and 48 h, and an equal volume of fresh release medium was supplemented. The drug concentration in the release medium was determined by HPLC, and the cumulative release amount was calculated.

Chromatographic conditions: Diamonsil C18 (250 mm × 4.6 mm, 5.0 μm) chromatographic column was used to detect the solution, with a column temperature of 30°C; the volume ratio of mobile phase acetonitrile-water solution was 30 to 70; the volume flow was 1 mL/min; UV detection wavelength was 306 nm; sample volume was 10 uL; detection time was 15 min. AWRK6 retention time was 5.2–7.5 min. The formula for calculating drug loading and encapsulation efficiency (LC) was as follows:

EE% = Wt/Ws × 100%

LC% = Wt/Wo × 100%

Note: Wt: the mass of AWRK6 contained in the nanoparticles; Wo: the initial dose of AWRK6; Ws: the total mass of the lyophilized nanoparticles.

### Neutralization of endotoxin by AWRK6/GO *in vitro*

According to the treatment method, the 96-well plate with 10 μL LPS (final concentration 0.2 ng/mL) added *in vitro* was divided into LPS group, AWRK6 (20 μg/mL) group, GO (20 μg/mL) group and AWRK6/GO (20 μg) /mL) group. 10 μL of the corresponding drug according to the group was added and incubated at 37°C for 30 min. An ELISA kit was employed to detect the residual LPS content in the plate.

### Detection of IL-8 levels in whole blood of mice

The ELISA method was used to detect the levels of IL-8 (SEKM-0046, Solarbio, China) and TNF-α (PT512, Beyotime, China) in the whole blood of mice. The blood from the heart of LPS mice in a heparinized tube was collected, 200 μL of heparinized mouse blood was added to a 96-well plate containing 25 μL LPS (final concentration 5 ng/mL), and 25 μL AWRK6 (20 μg/mL), GO (20 μg/mL) and AWRK6/GO (20 μg/mL) were added and incubated at 37°C for 24 hours and centrifuged at 1200 rmp/min for 8 minutes. The upper layer of plasma was analyzed by ELISA for the inflammatory factor IL- 8 levels. The animal study was reviewed and approved by Linyi People’s Hospital.

### Detection of TNF-α level in mouse macrophages

The ELISA method was used to detect the level of TNF-α in mouse peritoneal macrophages. The macrophages were inoculated in a 96-well plate at 2 × 105 cells/well, and 25 μL of AWRK6 (20 μg/mL), GO (20 μg/mL) and AWRK6/GO (20 μg/mL) and 25 μL LPS (final concentration 5 ng/mL) were added and incubated in a constant temperature incubator at 37°C for 6 hours; The ELISA method was used to detect the content of TNF-α in cells (PT512, Beyotime, China).

### Observation of the effect of AWRK6/GO on the survival rate of LPS mice

Construction of a mouse model of endotoxin sepsis: 0.5 mL of LPS with a concentration of 50 mg/kg was injected intraperitoneally into mice to construct a mouse model. LPS mice were randomly divided into LPS control group, AWRK6 group, GO group and AWRK6/GO group. The AWRK6, GO and AWRK6/GO groups were injected with AWRK6, GO or AWRK6/GO at 10 mg/kg, respectively. The survival status of the mice was recorded every 8 hours, and the observation was continued for 7 days.

### Biosafety evaluation of AWRK6/GO

The toxicity of AWRK6/GO in the liver cells, splenic lymphocytes and peritoneal macrophages of mice was determined by CCK-8 test. Mice hepatocytes, spleen lymphocytes and peritoneal macrophages (1 × 10^4^ cells/well) in the logarithmic growth phase were planted in 96-well plates, and AWRK6/GO (20 μg/mL) was added with concentrations of 10, 20, 30, 40 μM. After 24 hours, 10 μL of CCK-8 reaction solution was added to each well, and incubated in a constant temperature incubator for 4 hours. The absorbance at 450 nm on the microplate reader was detected.

The visceral tissues of mice injected with the drug AWRK6/GO for 20 days were examined by immunohistochemistry, and the pathological changes of the mice’s organs were observed. Immunohistochemical method: Mice injected with different drugs into the abdominal cavity were killed by cervical dislocation, and the heart, liver, spleen, lung and kidney tissues were collected. The tissues of mice were rinsed with PBS solution of pH 7.4, placed in 10% neutral formalin solution, and fixed at 25°C for 24 hours; the tissue samples were dehydrated by gradient concentration of ethanol, placed in xylene for 20 minutes until it became transparent, and embedded in paraffin at 60°C for 2 hours. The paraffin-embedded tissues were cut into sections with a thickness of 5 μm, extended in a 42°C water bath until they became transparent, loaded on a glass slide, heated an oven at 75°C for 1 hour and dewaxed with xylene till they became transparent; 100%, 95%, and 85% gradient concentration of ethanol was used for rehydration; hematoxylin was added dropwise onto the section for nuclear staining for 10 min, and rinsed with distilled water. After the sections were differentiated in hydrochloric acid-ethanol solution for 30s, they were rinsed again with distilled water and placed in 1% ammonia water to return to blue for 30 minutes; the sections were stained with 0.5% eosin staining solution for 3 minutes, rinsed with distilled water, dehydrated with 85%, 95% and 100% ethanol in sequence, and dealcoholized in xylene; neutral balsam was used for mounting.

### Cell lines

Mouse peritoneal macrophages were isolated from C57BL/6 mice aged 6 to 8 weeks. In short, sterile 3% thioglycollate medium (1 ml) was intraperitoneally injected after a 4-day prelavage with 10 mL PBS, the macrophages were then elicited by thioglycolate. Mouse hepatocytes and spleen lymphocytes were obtained following a reported protocol [39]. All cells were maintained in RPMI-1640 medium added with 10% FBS (Hyclone, USA), 100 U/mL penicillin and 100 U/mL streptomycin (Sigma, USA), and cultured in a humidified 37°C incubator filled with 5% CO_2_.

### Data analysis

The experiments involved in this study were carried out independently three times, and the data obtained were processed by SPSS 20.0 and graphed by GraphPad Prism 7. The measurement data were analyzed by variance analysis. A *t*-test was used for the difference between the two groups, and Bonferroni test was for the difference between multiple groups, recorded as the mean ± standard deviation. A difference was considered statistically significant when a *p* value was less than 0.05.
